# Supplementary material for: Simultaneous detection of Mycobacterium tuberculosis complex and resistance to Rifampicin and Isoniazid by MDR/MTB ELITe MGB® Kit for the diagnosis of tuberculosis
Source: PLoS One. 2020 May 4;15(5):e0232632. doi: 10.1371/journal.pone.0232632 (PMC7197853; doi:10.1371/journal.pone.0232632)
Supplement: Attachment — Submitted filename: Comments to the authors.docx [file pone.0232632.s001.docx]

**Comments to the authors:**

The retrospective study titled “Simultaneous detection of Mycobacterium tuberculosis complex and resistance to Rifampicin and Isoniazid by MDR/MTB ELITe MGB® Kit for the diagnosis of tuberculosis” by Bisognin F et al. demonstrated the use PCR based MDR/MTB ELITe MGB® Kit in the detection of Mycobacterium tuberculosis complex (MTBc). Timely detection is required for efficient management of TB cases word-wide. The diagnosis methods which can be operated in resource limited settings are required for efficient management of tuberculosis or disease caused by the MTBc. In this retrospective study the authors used the stored samples isolated from pulmonary, and extra-pulmonary sites. They compared their results with AFB staining, culture as well as Xpert MTB/RIF. Although, they showed good agreement in specificity and sensitivity with all the known methods used for the detection of MTBc in pulmonary and extra-pulmonary samples. To demonstrate their results in statistically significant and more reliable way the authors should generate ROC curve for their findings and include in the manuscript. This will accurately demonstrate their findings in statistically significant and meaning full way. The manuscript is well written and organized. The conclusions drawn are well supported by their data. The authors should have included more number of cases of smear negative samples. The authors mentioned MTBc through the manuscript. They should introduce few sentences describing species included. The manuscript could be accepted for publication after careful revision and addition of ROC analysis.
