# Supplementary material for: Simultaneous detection of Mycobacterium tuberculosis complex and resistance to Rifampicin and Isoniazid by MDR/MTB ELITe MGB® Kit for the diagnosis of tuberculosis
Source: PLoS One. 2020 May 4;15(5):e0232632. doi: 10.1371/journal.pone.0232632 (PMC7197853; doi:10.1371/journal.pone.0232632)
Supplement: Attachment — Submitted filename: Response to Reviewers Bisognin F et al.doc [file pone.0232632.s002.doc]

Bologna, 14^th^ April 2020

Manuscript Re-submission to PLOS ONE: Response to Reviewers (PONE-D-20-03632)

**Title**: Simultaneous detection of Mycobacterium tuberculosis complex and resistance to Rifampicin and Isoniazid by MDR/MTB ELITe MGB® Kit for the diagnosis of tuberculosis

**Authors**: Francesco Bisognin, Giulia Lombardi, Chiara Finelli, Maria Carla Re, Paola Dal Monte

We would like to thank you and the Reviewers for the useful comments provided. Based on all comments received, we have revised the original manuscript and added the ROC curve required.

The point-by-point responses to reviewers’ comments as well as the changes made to the original manuscript are described below.

Reviewers' comments:

Reviewer #1: Comments to the authors:

The retrospective study titled “Simultaneous detection of Mycobacterium tuberculosis complex and resistance to Rifampicin and Isoniazid by MDR/MTB ELITe MGB® Kit for the diagnosis of tuberculosis” by Bisognin F et al. demonstrated the use PCR based MDR/MTB ELITe MGB® Kit in the detection of Mycobacterium tuberculosis complex (MTBc). Timely detection is required for efficient management of TB cases word-wide. The diagnosis methods which can be operated in resource limited settings are required for efficient management of tuberculosis or disease caused by the MTBc. In this retrospective study the authors used the stored samples isolated from pulmonary, and extra-pulmonary sites. They compared their results with AFB staining, culture as well as Xpert MTB/RIF. Although, they showed good agreement in specificity and sensitivity with all the known methods used for the detection of MTBc in pulmonary and extra-pulmonary samples. To demonstrate their results in statistically significant and more reliable way the authors should generate ROC curve for their findings and include in the manuscript. This will accurately demonstrate their findings in statistically significant and meaning full way. The manuscript is well written and organized. The conclusions drawn are well supported by their data. The authors should have included more number of cases of smear negative samples. The authors mentioned MTBc through the manuscript. They should introduce few sentences describing species included. The manuscript could be accepted for publication after careful revision and addition of ROC analysis.

Many thanks for your comments.

As you suggested, we generated a ROC curve (Figure 1) to improve our statistical analysis. ROC analysis (AUC= 0.95) showed the high accuracy of the ELITe system for the diagnosis of tuberculosis.

Therefore we added the description of the statistical analysis used (in the Material and Method section), a paragraph in the Results section (lines 182-192), and a sentence in the Discussion section (lines 250-252).

Furthermore, as you suggested, we added a sentence describing MTB complex in the Introduction section (lines 49-51).

Reviewer #2: Current manuscript is technically sound and evaluated in a planned way. Statistical analysis is done appropriately.

Many thanks for your positive comments.

We hope that the revised manuscript can now be accepted for publication in PLOS ONE.

Thank you very much for your kind consideration.

Yours faithfully,

**Corresponding author:**

Dr. Giulia Lombardi, PhD

Department of Experimental, Diagnostic and Specialty Medicine - Microbiology Unit

University of Bologna - S. Orsola Malpighi University Hospital

Via Massarenti 9 - 40138 Bologna - Italy
